# Biofilm Analysis of Retrieved Dental Implants after Different Peri-Implantitis Treatments

**DOI:** 10.1155/2017/8562050

**Published:** 2017-04-09

**Authors:** Thaise C. Geremias, Juan F. D. Montero, Ricardo de Souza Magini, Guenther Schuldt Filho, Edival Barreto de Magalhães, Marco A. Bianchini

**Affiliations:** ^1^School of Dentistry, Center for Research on Dental Implants, Federal University of Santa Catarina, Florianópolis, SC, Brazil; ^2^School of Dentistry, UniSul University, Florianópolis, SC, Brazil

## Abstract

The aim of the current study was to analyse the planktonic growth of* Streptococcus mutans* on the surfaces of three implants retrieved after three different peri-implantitis treatments. Three implants from a male patient with high levels of bone loss were treated by mechanical debridement, chemical decontamination, and implantoplasty. After 4 months of follow-up, the implants were removed. The growth and biofilm formation were measured by spectrophotometry (OD_630 nm_) and scanning electron microscopy (SEM), after 48 hours of incubation. Results showed an average of* Streptococcus mutans* planktonic growth over the implants of 0.21 nm (mechanical debridement), 0.16 nm (chemical decontamination), and 0.15 nm (implantoplasty). Data were analysed by ANOVA and Tukey's test (*p* < 0.05 for chemical decontamination and implantoplasty). Implantoplasty and chemical decontamination showed the lowest levels of planktonic growth, indicating a possible influence of the modification procedures on the titanium surface on the initial biofilm attachment.

## 1. Introduction

In 2012, statements from the Estepona Consensus Meeting on peri-implantitis determined that “peri-implantitis is here defined as an infection with suppuration associated with clinically significant progressive crestal bone loss after the adaptive phase,” indicating oral biofilm within the indicative factors for developing the disease [1]. In 2016, it was concluded that the etiology of peri-implantitis is very complex. In fact, many risk indicators can explain the multicausality model, and peri-implantitis treatment protocols should be evaluated in relation to different risk factors and various implant features. It was established that plaque accumulation in dental implants triggers the inflammatory host response resulting in peri-implant mucositis/peri-implantitis [2].

The initial stages of biofilm formation by* S. mutans* (first 2 h following attachment) represent 60 to 80% of all primary colonizers that have different bacterial adhesins responsible for adhesion to the acquired pellicle. Quorum sensing is mediated by a competence stimulating peptide released upon exposure to low pH, posteriorly initiating a coordinated protective response [3].

In addition, dental implant surface irregularities facilitate the acquisition and maturation of biofilms [4]. Bacteria in the biofilm are dependent on entities coordinated and integrated metabolically. Microbial cells possess the ability to adhere to surfaces following four main steps: (I) random transport of bacterium to the surface, (II) initial adhesion (may be reversible), (III) strong adhesion to the surface by specific interactions, and (IV) colonization of the surface by multiple microbial cells and biofilm formation (maturation, growth, and structure of biofilms) [4, 5].

The removal of biofilm from the implant surface should be the main strategy through surface modification by decontamination, debridement, and removal of implant threads [6, 7]. The surface structure of presently available implant systems may have an impact on the progression of marginal bone loss [8]. The purpose of the present study was to analyse the planktonic growth of* Streptococcus mutans* on the surfaces of 3 implants retrieved after 3 different peri-implantitis treatments.

## 2. Case Report

A 54-year-old Caucasian male, without having presented a relevant past medical history, was referred to the Department of Implant Dentistry of the Federal University of Santa Catarina, Brazil, for peri-implantitis treatment in the area of implants 36, 45, and 46. After a submerged healing period of 6 months, cover screw exposure was noticed, associated with the presence of visible plaque formation combined with bleeding and suppuration on probing. Furthermore, cone-beam computed tomography showed a relevant peri-implant bone loss. All implants were external hex connection types (Neodent, Brazil) with equal dimensions (4.1 × 4 × 9 mm), manufactured with the dual acid etched technique.

The patient signed an informed consent and 3 different treatments were proposed:

- Implantoplasty (at the area of implant 36) was performed according to the Schwarz et al. protocol [9, 10].
- An open flap debridement and chemical decontamination using citric acid for two minutes (at the 45 area) were performed by the Khoury and Buchmann protocol [11].
- An open flap debridement (at the area of implant 46) was performed by the Schwarz et al. protocol [6].

**Figure 1:**
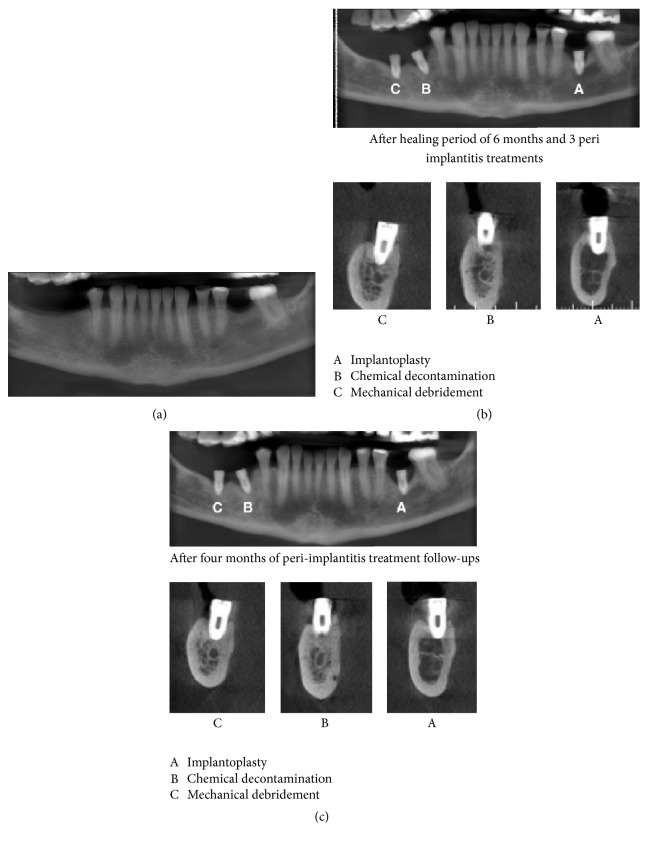
(a) Initial tomography, (b) 6 months' implant after placement and peri-implantitis treatments, (c) 4 months' after different peri-implantitis treatments. (A: implantoplasty, B: acid treatment, and C: debridement).

After four months of follow-up, tomography images exhibited an increase of bone loss, indicating failure of the proposed treatments (Figure 1).

**Figure 2:**
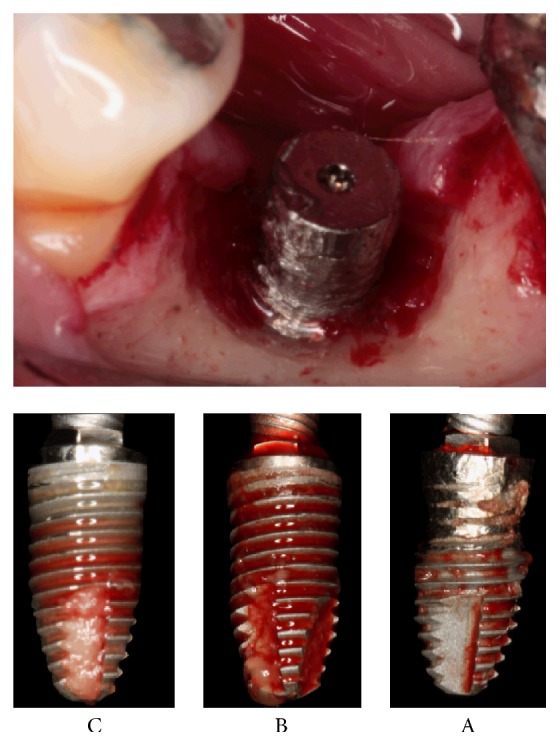
Peri-implantitis treatment. A: implantoplasty, B: acid treatment, and C: debridement.

Thus, followed by 4 months after different peri-implantitis treatments without an acceptable biological response, with the purpose of acquiring an accurate analysis, removal of the three implants was performed (Figure 2).

## 3. Biofilm Formation and Analysis

*S. mutans* ATCC 25175 was routinely grown under microaerophilic conditions for 48 h at 37°C in agar plates with 32 g/L of Brain Heart Infusion (BHI) (Bacto, Difco®, USA) and supplemented with 3 g/L of yeast extract and 200 g/L of sucrose (Bacto, Difco, USA). For the biofilm experiments,* S. mutans* cells were inoculated in Tryptic Soy Broth (TSB-Bacto, Difco, USA), supplemented with 3 g/L of yeast extract and 200 g/L of sucrose, and cultivated for 18 h at 37°C. After incubation, the cells were harvested by centrifugation at 5.000 rpm for 10 min at 4°C and were washed twice with a phosphate buffer solution (PBS). The bacterial pellet was then resuspended in TSB supplemented with mucin (2.5 g/L), peptone (5 g/L), urea (1 g/L), yeast extract (2 g/L), and sucrose (200 g/L) to obtain a suspension with an optical density (OD_630_) of 0.6, corresponding to 1 × 10^8^ CFU/mL. The OD_630_ readings were performed using a BioTek® spectrophotometer (USA). This cell suspension was used as the inoculum for the biofilm formation assays [12]. Implant samples were placed into 24-well plates, where each well contained 2 mL of* S. mutans* inoculum (1 × 10^8^ CFU/mL), and were incubated at 37°C. After 48 h of incubation, the planktonic growth was determined by OD_630_ readings as described above.

After biofilm formation, surfaces covered with biofilms were washed twice in PBS and fixed in 2% glutaraldehyde for 5 min. Next, the surfaces were washed three times in PBS and dehydrated through a series of graded ethanol solutions (50, 70, 80, 90, and 100%). Samples covered with* S. mutans* biofilms were sputter coated with gold and analysed by scanning electron microscope (JEOL JSM-6390LV, Japan) at 10 kV.

## 4. Statistical Analysis

For description of the data, mean values and SDs were calculated. Analysis of variance (ANOVA) was used to compare means among different groups of variables. One-way ANOVA followed by the Tukey's multiple range test were used to determine if there were significant differences between the planktonic growth in each experimental treatment, with a *p* value of <0.05.

## 5. Results

**Figure 3:**
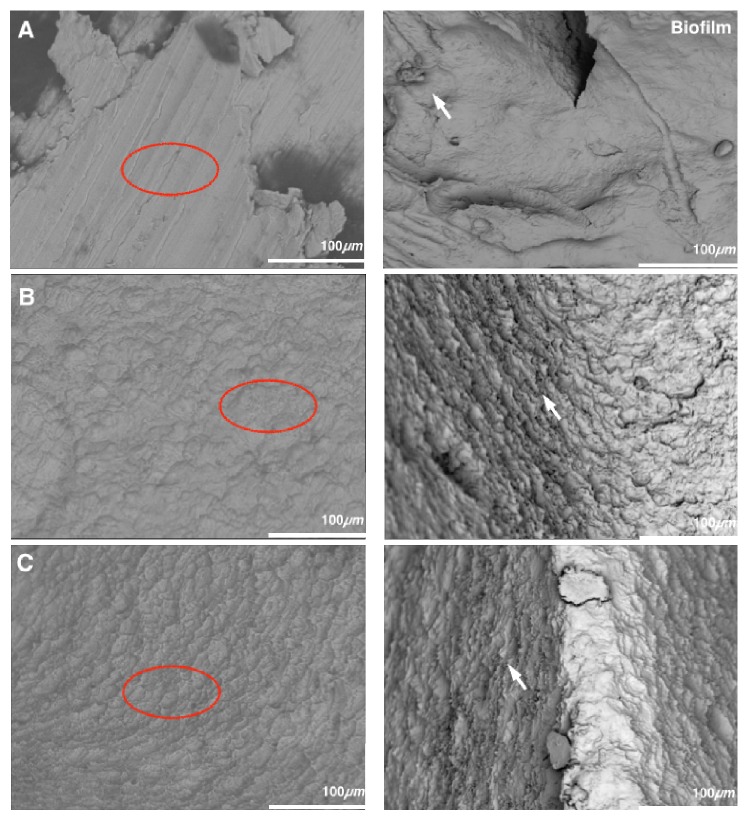
Scanning electron microscopy (SEM) images of the titanium surface displaying topography features before and after the* S. mutans* biofilm formation ((A) implantoplasty, (B) acid treatment, and (C) debridement) (Magnification D8.6 ×1.0k 100 *μ*m).

Scanning electron microscopy (SEM) examination of the implant surfaces before and after biofilm formation (Figure 3), where one correlated a distinct treatment for peri-implantitis, revealed surfaces with generalized asperities. The surface that underwent the implantoplasty process (Figure 3(A)) presented randomly oriented irregularities, which were classic features of a titanium surface that suffered from the modification process to achieve a moderately roughened surface. Additionally, the acid treatment surface (Figure 3(B)) presented peaks and valleys without continuity in contrast with the debridement process (Figure 3(C)) which exhibited peaks and valleys with continuity. Nonetheless, the biofilm formation was evidenced in all treatment surfaces without exception where colonies grew widely separated.

The process of biomass formation on peri-implantitis treatment surfaces occurred after 48 hours of incubation with* S. mutans*.

**Figure 4:**
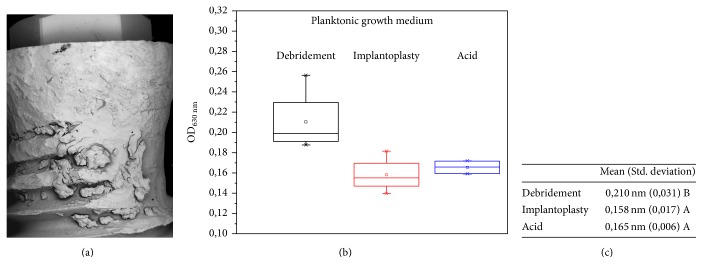
Biofilm formation on implantoplasty treatment surface (a). S.* mutans* planktonic growth in implants for 48 h in TSB (37°C), measured by spectrophotometer with an optical density of 630 nm (b) and table exhibiting mean values, according to the specific treatment (c).

Furthermore, the planktonic growth absorbance measurements by spectrophotometry after 48 h in TSB (37°C) (Figure 4(b)) also observed a higher tendency for planktonic growth on the sample during the debridement treatment (0.21 nm), when compared to the acid treatment (0.16 nm) and implantoplasty (0.15 nm) (Figures 4(b) and 4(c)). There were statistical differences (*p* < 0.05) for the chemical decontamination and implantoplasty when compared with the mechanical debridement.

## 6. Discussion

Several studies currently seek knowledge regarding the results of treatments made on dental implants, as well as clearly understanding the conditions and structure of the implants in function and subsequent loss. This can identify a correlation between the composition, dimensions, surface treatments, and risk factors in the future diagnosis of the disease [13].

The present study focused on describing modifications towards the influence of biofilm formation of each specific treatment, whilst sharing the same fundamental purpose of disrupting the biofilm development [14].

Surface microtopography is an important influence in biofilm formation and bacterial colonization. In 2012, the Almaguer-Flores et al. study found a correlation and influence of the microtopography and hydrophilicity of titanium (Ti) substrates on initial oral biofilm formation, concluding that the initial biofilm formation and composition were affected by the microtopography and hydrophilicity of the surface [15].

In the 2015, Di Giulio et al. showed that the modification of the titanium surfaces can influence the colonization and biofilm formation of* Porphyromonas gingivalis*; the surface treatment overcomes the differences in the material composition [16]. In addition, the difference in titanium surface roughness was associated with variations in the antifungal resistance of the candida biofilm [17].

A large number of treatments for peri-implantitis are available in the literature, with the aim towards thorough cleaning of the contaminated surface and understanding that the removal of biofilm from the implant surface is a priority [18, 19].

The present study evidences that the surface modification of implants affected by peri-implantitis, either by implantoplasty or chemical decontamination, favors a lower accumulation of biofilm formation (*S. mutans*) than just with mechanical debridement.

## 7. Conclusions

*S. mutans* planktonic growth on distinct peri-implantitis treatment surfaces confirmed a notable influence of the modification procedures on the titanium surface in the initial biofilm attachment. Notwithstanding, additional studies involving multispecies biofilms and additional clinical trials are needed to confirm the effect of surface modifications for treatment of peri-implantitis.
